# Haptoglobin genotype and its relation to asymptomatic cerebral small-vessel disease in type 1 diabetes

**DOI:** 10.1007/s00592-023-02059-2

**Published:** 2023-03-01

**Authors:** M. I. Eriksson, A. Syreeni, N. Sandholm, E. H. Dahlström, D. Gordin, T. Tatlisumak, J. Putaala, Per-Henrik Groop, J. Martola, L. M. Thorn, The FinnDiane Study Group

**Affiliations:** 1 Folkhälsan Institute of Genetics, Folkhälsan Research Center, Helsinki, Finland; 2 Department of Nephrology, University of Helsinki and Helsinki University Hospital, Biomedicum Helsinki, Haartmaninkatu 8, 00290 Helsinki, Finland; 3 Research Program in Clinical and Molecular Metabolism, University of Helsinki, Helsinki, Finland; 4 Joslin Diabetes Center, Harvard Medical School, Boston, MA USA; 5 Minerva Institute for Medical Research, Helsinki, Finland; 6 Neurology, University of Helsinki and Helsinki University Hospital, Helsinki, Finland; 7 Department of Neurology, Sahlgrenska University Hospital, Gothenburg, Sweden; 8 Department of Clinical Neuroscience/Neurology, Institute of Neurosciences and Physiology, Sahlgrenska Academy at University of Gothenburg, Gothenburg, Sweden; 9 Department of Diabetes, Central Clinical School, Monash University, Melbourne, VIC Australia; 10 Department of Clinical Neuroscience, Karolinska University Hospital, Karolinska Institute, Stockholm, Sweden; 11 Department of Radiology, University of Helsinki and Helsinki University Hospital, Helsinki, Finland; 12 Department of General Practice and Primary Health Care, University of Helsinki and Helsinki University Hospital, Helsinki, Finland

**Keywords:** Diabetes mellitus, Type 1 diabetes, Cerebral small-vessel disease, Brain MRI, Stroke, Haptoglobin genotype

## Abstract

**Aim:**

Cerebral small-vessel disease (SVD) is prevalent in type 1 diabetes and has been associated with the haptoglobin variant allele Hp1. Contrarily, the Hp2-allele has been linked to cardiovascular disease and the role of haptoglobin-genotype in asymptomatic SVD is unknown. We, therefore, aimed to evaluate the alleles’ association with SVD.

**Methods:**

This cross-sectional study included 179 neurologically asymptomatic adults with type 1 diabetes (women 53%, mean age 39 ± 7 years, diabetes duration 23 ± 10 years, HbA_1c_ 8.1 ± 3.2% [65 ± 12 mmol/mol]). Examinations included genotyping (genotypes Hp1-1, Hp2-1, Hp2-2) by polymerase chain reaction, clinical investigation, and magnetic resonance brain images assessed for SVD manifestations (white matter hyperintensities, cerebral microbleeds, and lacunar infarcts).

**Results:**

SVD prevalence was 34.6%. Haptoglobin genotype frequencies were 15.6% (Hp1-1), 43.6% (Hp1-2), and 40.8% (Hp2-2). Only diastolic blood pressure differed between the genotypes Hp1-1, Hp1-2, and Hp2-2 (81 [74–83], 75 [70–80], and 75 [72–81] mmHg, *p* = 0.019). Haptoglobin genotype frequencies by presence versus absence of SVD were 16.1%; 46.8%; 37.1% versus 15.4%; 41.9%; 42.7% (*p* = 0.758). Minor allele frequencies were 39.5% versus 36.3% (*p* = 0.553). Hp1 homozygotes and Hp2 carriers displayed equal proportions of SVD (35.7% vs 34.4%, *p* > 0.999) and SVD manifestations (white matter hyperintensities 14.3% vs 17.9%, *p* = 0.790; microbleeds 25.0% vs 21.9%, *p* = 0.904; lacunar infarcts 0% vs 3.6%, *p* > 0.999). Hp1-1 was not associated with SVD (OR 1.19, 95% CI 0.46–2.94, *p* = 0.712) when adjusting for age, blood pressure, and diabetic retinopathy.

**Conclusions:**

Although the SVD prevalence was high, we detected no significant association between SVD and haptoglobin-genotype.

**Supplementary Information:**

The online version contains supplementary material available at 10.1007/s00592-023-02059-2.

## Introduction

Cerebrovascular disease is among the most common causes of disability worldwide [1]. In type 1 diabetes, the risk of cerebrovascular disease, particularly stroke, is fourfold compared to the general population [2] and the proportion of strokes attributable to cerebral small-vessel disease (SVD) is as substantially increased [3, 4]. Aside from overt disease, also in stroke-free individuals, SVD is more common in type 1 diabetes compared to healthy controls [5].

SVD affects the microvasculature of the brain and is visible on magnetic resonance images (MRI) by its manifestations, which include white matter hyperintensities, cerebral microbleeds, lacunar infarctions, and cortical superficial siderosis [6]. Although common in type 1 diabetes, SVD remains scarcely studied [5]. Within the Finnish Diabetic Nephropathy (FinnDiane) Study, we have found increased blood pressure [5, 7], carotid intima-media thickness [8] and the severity of diabetic retinopathy [9] to be associated with SVD in individuals with type 1 diabetes. Other studies have likewise linked SVD with arterial stiffness [10] and diabetic retinopathy [11]. In addition, SVD has been suggested to associate with the haptoglobin (Hp) genotype [12].

Hp is a plasma protein that binds to free haemoglobin, thereby affecting its oxidative activity. The protein is affected by genetic variation. Two variant alleles, Hp1 and Hp2 give rise to three genotypes Hp1-1, Hp2-1, and Hp2-2, of which Hp2-2 possesses the weakest antioxidative properties [13] and has been linked to an increased risk of cardiovascular disease [14, 15]. Consistent observations of this association, however, mostly regard studies including individuals with type 2 diabetes [16, 17]. In type 1 diabetes, Hp2-2 has been associated with an increased risk of coronary artery disease [18], kidney disease [19], and cardio-renal mortality [20].

On the other hand, also the less-frequently observed genotype Hp1-1 has been linked to cerebrovascular disease in the general population [21]. In type 1 diabetes Hp1-1 has been linked to stroke in hypertensive individuals [22], and white matter hyperintensities [12]—a non-acute manifestation of SVD. Nevertheless, when studied in the Finnish general population, the Hp1-1 genotype was not associated with any subtype of ischemic stroke, including subtypes attributable to small-vessel occlusion i.e. SVD [23]. Moreover, in our recent large study, we found no association between haemorrhagic or ischemic stroke and Hp-genotype in our cohort of individuals with type 1 diabetes, including 500 stroke cases and 3,806 controls [24].

Although not known, the impact of a distinct genotype could vary for different cardiovascular outcomes, such that Hp2-2 relates to atherosclerosis and large-vessel disease, whereas Hp1-1 to SVD. It is yet unknown whether the Hp-genotype is associated with the prevalence of SVD, or if a particular manifestation occurs more often in certain genotypes. We, therefore, aimed to elucidate whether the Hp-genotype is associated with SVD, or any of its manifestations, in our cohort of 179 neurologically asymptomatic individuals with type 1 diabetes.

## Materials and methods

Within the nationwide FinnDiane Study, we initiated a sub-study at the Helsinki University Hospital (HUS) study centre in 2010. This sub-study aims to identify early risk factors for cerebrovascular disease in type 1 diabetes. The FinnDiane study [25] and the protocol of the aforementioned sub-study have been described in detail previously [5].

Individuals entering the HUS study centre were consecutively recruited for a study visit that included a brain MRI if they met the inclusion criteria: age 18–50 years and type 1 diabetes onset before 40 years of age. Exclusion criteria were kidney failure (dialysis or kidney transplantation), previous clinical signs of cerebrovascular disease, and contraindications for MRI. A total of 191 participants were enrolled and the Hp-genotype was successfully determined for 179 (93.7%) participants, all eligible for this study. With this sample size and significance level (α = 0.05) we were able to detect an effect of OR = 1.91 with 0.80 power.

All participants entered a study visit. Their medical history was evaluated thoroughly, and data were verified from the participant’s medical records. Anthropometrics, office blood pressure, and current medication were recorded. Body mass index was calculated by dividing body mass by the square of the body height. Participants furthermore filled in questionnaires on lifestyle, such as smoking. Diabetic retinopathy was verified and categorized based on fundus images with “any retinopathy” indicating the presence of at least retinal microaneurysms, and proliferative diabetic retinopathy indicating neovascularization or previous treatment with retinal photocoagulation.

Glycated haemoglobin (HbA_1c_), haemoglobin, creatinine, total cholesterol, LDL-cholesterol, HDL-cholesterol, and triglycerides were determined from blood samples. The glomerular filtration rate was estimated using the Chronic Kidney Disease Epidemiology Study (CDK-EPI)-formula [26]. Albumin excretion rate was determined through 24-h urine collection and participants with a urinary albumin excretion rate of ≥ 20 µg/min or ≥ 30 mg/24 h in two out of three urine collections were considered as having albuminuria. All blood and urine samples were analysed in the central laboratory of Helsinki University Hospital.

DNA was extracted from white blood cells. We used a method similar to Ijäs et al. [23] for Hp genotyping. Briefly, DNA was amplified with two polymerase chain reactions (PCRs), and two pairs of primers (Supplementary Table 1) were used to generate DNA strings of distinct length, depending on the presence versus absence of exons 5 and 6 (i.e., Hp2 vs Hp1). DNA fragments were analysed in agarose gel electrophoresis or with Caliper LabChip GX Instrument (PerkinElmer, MA, USA) at the Finnish Institute of Molecular Medicine to determine the Hp-genotype.

Brain-MRI was performed at the Helsinki Medical Imaging Center, Helsinki University Hospital, with a 3.0 Tesla scanner (Achieva, Philips, Best, The Netherlands). Images were assessed for markers of SVD by an experienced neuroradiologist (J.M.). As markers, we considered white matter hyperintensities (a score of ≥ 1 on the Fazekas scale), cerebral microbleeds, lacunar infarctions, and superficial siderosis, based on standardised criteria [27]. The neuroradiologist was blinded to all clinical data. Brain-MRI and assessment of the images have been described previously in further detail [5]. For analysis, a participant with a positive finding of any marker was considered to display SVD. The different markers were also analysed categorically as separate manifestations of SVD, i.e., presence versus absence of the specific manifestation.

Continuous parametric variables were analysed using one-way ANOVA, with central tendency presented as mean ± standard deviation. For analysis of non-parametric variables, the Kruskal–Wallis test was used with results presented as median (interquartile range). Categorical variables were compared using the chi-squared test, and Fisher’s exact test when observed frequencies were < 5.

To analyse the relationship between SVD and genotype Hp1-1, multiple logistic regression analysis was used. SVD was analysed as the dependent variable, with Hp-genotype and clinical confounders as independent variables. Clinical confounders were chosen based on their significance in univariable analysis. The analyses of SVD (any manifestation), and all its manifestations, were performed in separate models. Results are presented as odds ratios (OR) with 95% confidence interval (CI). A *p* value < 0.05 was considered statistically significant.

Power analyses were performed post hoc to evaluate the smallest detectable effect size in the cohort. The smallest detectable OR for SVD as a binary outcome (present vs absent) was calculated based on minor allele frequency, the proportion of SVD cases, and the number of participants using the R genpwr package version 1.0.4 [28]. A significance level α = 0.05 was chosen for the analysis. All analyses were performed with R version 4.0.0.

## Results

The study participants had a mean age of 38.8 ± 7.3 years, 95 (53.1%) were women, participants had a mean diabetes duration of 23.3 ± 10.1 years, and their mean HbA_1c_ was 8.1 ± 3.5% (65 ± 12 mmol/mol). None of the participants had peripheral arterial disease. A total of 62 (34.6%) participants had SVD. Cerebral microbleeds were observed in 40 (22.3%), white matter hyperintensities of Fazekas ≥ 1 in 31 (17.3%), and lacunar infarctions in four (2.2%). None of the participants had cortical superficial siderosis. White matter hyperintensities and cerebral microbleeds were observed simultaneously in nine participants. All four participants with lacunar infarctions had some additional manifestation: two had white matter hyperintensities and the other two had cerebral microbleeds.

A total of 28 (15.6%) participants had the Hp1-1, 78 (43.6%) the Hp 2-1, and 73 (40.8%) the Hp2-2 genotype. The distribution was within the Hardy–Weinberg equilibrium (*p* = 0.647) and similar to frequencies previously observed in the Finnish population [23]. The minor allele frequency within the study cohort was 37.4%. Clinical characteristics by genotype are presented in Table 1. Except for median diastolic blood pressure, participants with different genotypes did not differ in clinical characteristics (Table 1).

**Table 1:** Clinical characteristics by haptoglobin genotype

	Hp1-1	Hp2-1	Hp2-2	*p* value
N	28	78	73	
Sex (women), n (%)	19 (67.9)	41 (52.6)	35 (47.9)	0.198
Age, years	38.5 ± 6.4	38.4 ± 7.9	39.2 ± 6.9	0.792
Diabetes duration, years	22.2 ± 7.8	23.8 ± 10.7	23.2 ± 10.2	0.794
Age at diabetes onset, years	14.7 (8.1–24.8)	12.3 (7.6–20.7)	14.2 (8.9–21.9)	0.563
Body mass index, kg/m^2^	27.4 ± 4.2	27.1 ± 4.24	25.8 ± 3.7	0.089
Systolic blood pressure, mmHg	132 ± 15	131 ± 14	129 ± 15	0.523
Diastolic blood pressure, mmHg	81 (74–83)	75 (70–80)	75 (72–81)	0.019
Antihypertensive medication, n (%)	14 (50.0)	28 (35.9)	20 (27.4)	0.097
HbA_1c_, % [mmol/mol]	8.1 (7.6–8.6) [64 (60–70)]	8.1 (7.3–8.7) [65 (56–72)]	8.2 (7.4–8.8) [66 (57–73)]	0.540
Haemoglobin, g/l	139 (133–144)	137 (128–146)	139 (130–145)	0.936
Total cholesterol, mmol/l	4.4 (4.1–5.4)	4.4 (4.0–5.0)	4.5 [4.0–4.9)	0.770
LDL cholesterol, mmol/l	2.6 (1.9–3.0)	2.3 (2.1–2.7)	2.5 (2.0–3.0)	0.456
HDL cholesterol, mmol/l	1.5 ± 0.4	1.5 ± 0.4	1.6 ± 0.4	0.854
Triglycerides, mmol/l	0.9 (0.7–1.5)	1.0 (0.7–1.5)	0.9 (0.6–1.2)	0.254
Lipid-lowering medication, n (%)	5 (17.9)	17 (21.8)	15 (20.5)	0.907
Creatinine, µmol/l	66 (59–78)	71 (62–82)	67 (61–78)	0.346
eGFR, ml/min/1.73 m^2^	108 (96–113)	106 (95–114)	109 (97–115)	0.538
Albuminuria, n (%)	5 (17.9)	14 (17.9)	11 (15.1)	0.881
History of smoking, n (%)	11 (39.3)	20 (25.6)	26 (35.6)	0.276
Any diabetic retinopathy, n (%)	27 (96.4)	61 (79.2)	57 (79.2)	0.094
Proliferative diabetic retinopathy, n (%)	6 (21.4)	12 (15.4)	9 (12.3)	0.517
Coronary artery disease, n (%)	0 (0.0)	1 (1.3)	0 (0.0)	0.521

In Table 1, results are presented as n (%), average ± standard deviation, and median (quartiles) with *p* values given for comparison across all groups. Continuous parametric variables have been analysed with one-way ANOVA, non-parametric variables with the Kruskal–Wallis test, and categorical variables with the chi-squared test or Fisher’s exact test when frequencies were < 5. HbA_1c_ indicates glycated haemoglobin and eGFR estimated glomerular filtration rate

**Fig. 1:**
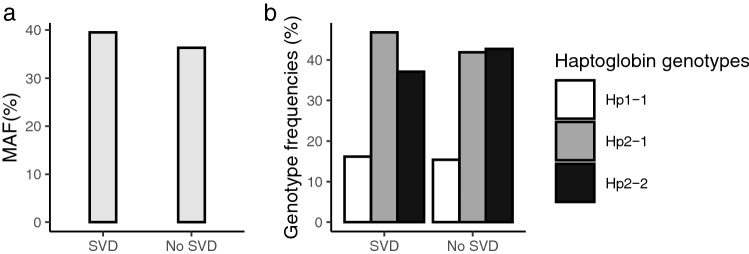
Allele and genotype frequencies in individuals with versus without cerebral small-vessel disease (SVD). **a** displays minor allele frequency (MAF) in individuals with (MAF 39.5%) versus without SVD (MAF 36.3%). **b** displays frequencies of genotypes Hp1-1 (16.1 vs 15.4%), Hp1-2 (46.8 vs 41.9%) and Hp2-2 (37.1 vs 42.7%) in SVD versus no SVD

Figure 1 illustrates genotype frequencies and minor allele frequencies for participants with versus without SVD. When analysing genotype frequencies by presence versus absence of SVD with the chi-square test, the difference in genotype frequencies was not statistically significant (*p* = 0.758). Likewise, minor allele frequencies by presence versus absence of SVD did not differ between the groups (*p* = 0.553). Also, when analysing the additive effect of the Hp1 alleles by simple logistic regression, we did not detect a significant association between SVD and the number of Hp1 alleles (OR 1.14 95% CI 0.73–1.75, *p* = 0.566).

We tested for differences in the prevalence of separate SVD markers across genotypes, by using the chi-squared test. Results are presented in Table 2. The prevalence of white matter hyperintensities did not vary significantly across different genotypes (Table 2). The same was true for cerebral microbleeds and lacunar infarctions (Table 2). Additionally, we were not able to detect a difference between minor allele frequencies in individuals with versus without the respective manifestations: cerebral microbleeds 37.4% versus 37.5%, *p* = 0.988, and white matter hyperintensities 40.3% versus 36.8%, *p* = 0.605. As we had only four observations of lacunar infarctions, we could not perform statistical testing for those.

**Table 2:** Prevalence of cerebral small-vessel disease by genotype

	Hp1-1	Hp2-1	Hp2-2	*p* value
N	28	78	73	
Cerebral small-vessel disease (any manifestation)	10 (35.7)	29 (37.2)	23 (31.5)	0.758
White matter hyperintensities	4 (14.3)	17 (21.8)	10 (13.7)	0.428
Cerebral microbleeds	7 (25.0)	16 (20.5)	17 (23.3)	0.860
Lacunar infarctions	0 (0)	1 (1.3)	3 (4.1)	0.563

In Table 2, results are presented as n (%), and variables have been analysed with the chi-squared test or Fisher’s exact test when frequencies were < 5

In further analysis, homozygotes for Hp1 (N = 28) were compared to Hp2 carriers (Hp2-1 or Hp2-2, N = 151). Except for diastolic blood pressure (Hp1-1 average 80 ± 6 mmHg vs Hp2 carriers 76 ± 9 mmHg, *p* = 0.027), homozygotes for Hp1 did not significantly differ from the rest of the participants regarding clinical characteristics (data not shown).

Table 3 presents observations of cerebral SVD in individuals with genotype Hp1-1 versus Hp2 carriers. The SVD prevalence did not vary significantly between Hp1 homozygotes and Hp2 carriers (Table 3). Furthermore, genotype Hp1-1 was not associated with SVD after adjustment for age, systolic and diastolic blood pressure, and any diabetic retinopathy in a logistic regression model (Table 3). Neither did we detect an association between Hp1-1 and white matter hyperintensities or cerebral microbleeds when analysing each manifestation separately, adjusted for the same clinical confounders (Table 3).

**Table 3:** Association between cerebral small-vessel disease and haptoglobin genotype Hp1-1

	Unadjusted analysis			Adjusted model		
	Hp1-1	Hp2 carriers	*p* value	OR	95% CI	*p* value
N	28	151				
Cerebral small-vessel disease, n (%)	10 (35.7)	52 (34.4)	> 0.999	1.19	0.46–2.94	0.712
White matter hyperintensities, n (%)	4 (14.3)	27 (17.9)	0.790	0.79	0.21–2.44	0.703
Cerebral microbleeds, n (%)	7 (25.0)	33 (21.9)	0.904	1.23	0.43–3.25	0.679
Lacunar infarctions, n (%)	0 (0.0)	4 (2.6)	> 0.999	–	–	–

*p* values for unadjusted analysis are calculated with chi-square testing from univariable analyses and results are presented as n (%). In adjusted analysis, Hp1-1 compared to Hp2 carriers, are analysed in a logistic regression model adjusted for age, systolic and diastolic blood pressure, and any diabetic retinopathy. Results are presented as odds ratios (OR) with 95% confidence intervals (CI)

## Discussion

In this study, we did not detect a significant association between Hp-genotype and asymptomatic SVD, despite a rather high prevalence of the disease among participants. This negative finding extends to our recent negative findings of Hp-genotype and risk of stroke in a large type 1 diabetes cohort [24]. To our knowledge, this is the first study to examine the Hp-genotype in relation to all manifestations of SVD, that is*,* microbleeds, white matter hyperintensities, and lacunar infarctions in type 1 diabetes.

Our findings differ from previous type 1 diabetes studies which have demonstrated an association between white matter hyperintensities and the Hp1 allele. In a study by Costacou et al. [12], including 94 individuals, they found individuals of genotype Hp1-1 to have greater white matter hyperintensities, and that Hp contributed significantly to white matter hyperintensity-variation in the corpus callosum, but not white matter hyperintensities in the total brain volume.

In contrast to Costacou et al. [12], we did not assess the localization or volume of white matter hyperintensities, rather, white matter hyperintensities were quantified according to the Fazekas scale for analysis [29]. Moreover, compared to our study, Costacou et al. had a very high prevalence of white matter hyperintensities (99% compared to 17% in our study). Their participants were older than ours, had an almost 20 years longer duration of diabetes and, additionally, had an earlier onset of the disease. Even though we did not observe an association between the duration of diabetes and white matter hyperintensities in our previous study [5], the difference in these clinical characteristics might account for some of the divergence between the studies. In addition, Costacou et al. do not assess cerebral microbleeds, which were the most common manifestation of our study, frequently coinciding with white matter hyperintensities [12].

Our results, however, agree with previous studies in individuals without diabetes, where no association between Hp-genotype and cerebral SVD were reported [23, 30]. Nevertheless, findings have been contradictory also in individuals without diabetes and one previous study linked the genotype Hp1-1 to larger volumes of white matter hyperintensities [31]. Reports on the association between Hp-genotype and lacunar infarctions are likewise contradictory [21, 30], while, to the best of our knowledge, no studies on cerebral microbleeds exist.

While the role of the Hp-genotype in humans, in vivo*,* is still unclear, studies in animal models and in vitro have described potential pathophysiological roles of Hp in both cerebral haemorrhages [32] and ischemia [33] previously. On the one hand, Hp1-1 has been linked to reduced endothelial repair and is suggested to induce ischemic damage, particularly lacunar strokes, in SVD [33]. On the other hand, along with endothelial dysfunction, inflammation is a central component of SVD [34, 35] and Hp2-2 has been suggested to promote inflammation after an acute haemorrhagic brain event [36]. One could hypothesize that the role of a certain Hp-genotype may vary depending on the type of tissue damage. Whereas one genotype might lessen ischemic damage, another may protect from vessel leakage, including microbleeds. The association between Hp1-1 and SVD could, therefore, be limited to certain manifestations, such as white matter hyperintensities, as observed by Costacou et al. [12]. Nevertheless, we did not detect any significant associations between a specific manifestation and Hp-genotype among our participants.

Possibly, a potential association between Hp-genotype SVD could become more prominent as the disease advances. Previously reported associations have, indeed, been observed in more severe SVD, e.g., overt lacunar infarction and a larger extent of white matter hyperintensities [12, 21, 31]. In our study, participants were asymptomatic and MRI findings were moderate (none of the participants had a Fazekas score > 2). Therefore, we were not able to evaluate the severity of SVD in our participants.

Some studies have suggested that the association between SVD and Hp1-1 is more pronounced in individuals with hypertension [22, 31]. One could speculate that hypertension would amplify the impact of Hp1-1 through mechanisms of impaired endothelial repair as previously described [33]. Of note is, that in our study participants with the Hp1-1 genotype had higher diastolic blood pressure than the Hp2 carriers and, though not significant, there was a trend towards more frequent use of antihypertensive medication in the Hp1-1 group. In the logistic regression model, we adjusted for blood pressure, however, due to the low number of individuals with hypertension (62 participants used antihypertensive medication) we were not able to perform further analysis stratified by hypertension.

A potential influence of survival bias has been acknowledged in some previous studies examining the Hp-genotype in SVD [12]. Survival bias is possible in our study as well, as excluding individuals with a previous cerebrovascular event or kidney failure could have masked a competing risk. Even so, considering that Hp2-2 has consistently been associated with cardiovascular and kidney disease in diabetes [16, 19, 20], such a selection would likely have led to an overestimation of the effect of Hp1. Yet, we did not observe a significant association between the Hp1 allele and SVD.

A limitation of this study is that the statistical power does not allow us to reject the possibility of a subtle association between Hp-genotype and SVD. However, to the best of our knowledge, this is the largest study with brain-MRI and Hp genotyping in type 1 diabetes to date. Our statistical power analysis showed that effect sizes of OR 1.91 or larger would be detectable with 80% probability (α = 0.05) for this number of participants, indicating that the study is well-powered to detect more prominent associations. Of note is, that although we would have been able to detect larger effects (OR > 1.91), the sample size required to detect more subtle effects (OR < 1.5) with a probability of 0.8 is 4,295 participants (α = 0.05). Regardless, we consider the standardised brain MRI and the detailed assessment of the images to be a substantial strength of our study. Another strength of our study is the unique and well-characterized study population.

## Conclusions

Our results suggest that there is no prominent association between asymptomatic SVD and genetic variation in Hp. Although our current study has a unique cohort with both MRI and Hp-genotype data of individuals with type 1 diabetes, and we have previously presented similar observations for stroke, i.e. overt disease, our results highlight a call for more comprehensive studies on the topic of SVD in type 1 diabetes.

## Supplementary Information

Below is the link to the electronic supplementary material.

Supplementary file1 (PDF 105 kb)

## Data Availability

Individual-level data for the study participants are not publicly available because of the restrictions due to the study consent provided by the participant at the time of data collection.

## Abbreviations

SVD: Cerebral small-vessel disease
MRI: Magnetic resonance imaging
Hp: Haptoglobin
HbA_1c_: Glycated haemoglobin
