# Supplementary material for: Haptoglobin genotype and its relation to asymptomatic cerebral small-vessel disease in type 1 diabetes
Source: Acta Diabetol. 2023 Mar 1;60(6):749–56. doi: 10.1007/s00592-023-02059-2 (PMC10148779; doi:10.1007/s00592-023-02059-2)
Supplement: Supplementary file 1 — Supplementary file1 (PDF 105 kb) [file 592_2023_2059_MOESM1_ESM.pdf]

Supplementary Materials

Supplementary table 1: Haptoglobin genotyping

	1st PCR			2nd PCR		
Forward primer 5′-3′	GAGGGGAGCTTGCCTTTCCATTG			CAGAACCAGAGGCAAAGACC		
Reverse primer 5′-3′	GAGATTTTTGAGCCCTGGCTGGT			AACCGAGTGCTCCACATAGC		
1×PCR mix	12 µl	Platinum PCR Supermix High Fidelity (Invitrogen)		4 µl	5×Phusion buffer HF (4 mM) (Thermo Fisher)	
				8.8 µl	H_2O	
				1 µl	dNTPs (4 mM)	
				0.2 µl	Phusion Hot Start II (2 U/µl)	
	0.11 µl	Forward primer (20 µM)		1 µl	Forward primer (10 µM)	
	0.11 µl	Reverse primer (20 µM)		1 µl	Reverse primer (10 µM)	
Genomic DNA	8 µl	Genomic DNA (2.5 ng/µl)		4 µl	Genomic DNA (2.5 ng/µl)	
Total 1×PCR volume	20 µl			20 µl		
PCR program	94°C	2 min		98°C	30 s	
	94°C	20 s	×30 cycles	98°C	10 s	×35 cycles
	55°C	20 s		64°C	10 s	
	68°C	3 min 30 s		72°C	15 s	
	68°C	7 min		72°C	10 min	
	8°C	∞		4°C	∞	
Amplicon size	1,757 bp à Hp1-1<br>1,757 bp and 3,481 bp à Hp2-1<br>3,481 bp à Hp2-2			No product à Hp1-1<br>442 bp à Hp2-1 or Hp2-2		

Supplementary table 1 presents the protocol of Hp-genotype determination by polymerase chain reaction (PCR). DNA was amplified with two PCRs, and two pairs of primers were used (forward primer and reverse primer) to generate DNA strings of distinct length (amplicon size), depending on the presence vs absence of alleles Hp2 vs Hp1.
